# Effects of Black Soldier Fly ( *Hermetia illucens* ) Larvae on Bulk Secondary Metabolite Levels and Antioxidant Activity in *Moringa oleifera* Biomass

**DOI:** 10.1002/pei3.70163

**Published:** 2026-05-13

**Authors:** Teh Exodus Akwa, Honorine Ntangmo Tsafack, Tchuenguem Tchuenteu Roland, Tenkeu Armel Cyrille, Lucy Gitau, Godfroy Rostant Pokam Djoko, Tchuinkam Timoléon, Leon Tapondjou Azefack

**Affiliations:** ^1^ Research Unit of Biology and Applied Ecology, Department of Animal Biology, Faculty of Science University of Dschang Dschang Cameroon; ^2^ Faculty of Agronony and Enviromental Sciences Evangelical University of Cameroon Bandjoun Cameroon; ^3^ Research Unit of Environmental and Applied Chemistry, Faculty of Science, Department of Chemistry University of Dschang Dschang Cameroon

**Keywords:** antioxidant activity, *Hermetia illucens*, larval bioconversion, *Moringa oleifera*, secondary metabolites

## Abstract

*Moringa oleifera* is a nutrient‐rich tropical plant containing bioactive secondary metabolites. While these metabolites provide antioxidant and antimicrobial properties, they may reduce digestibility or palatability when used as animal feed. This study evaluated the impact of Black soldier fly (BSF) larvae on the bulk levels of secondary metabolites and antioxidant activity in *M. oleifera* leaves. Leaves were incubated with 1500 7‐day‐old BSF larvae at 27°C and 65% relative humidity for 16 days, with three replicates per treatment. Post‐treatment material, consisting of residual substrate and larval excreta (frass), was analyzed using qualitative phytochemical screening and quantitative spectrophotometric assays. Larval treatment was associated with significantly lower levels of total phenols (0.712 ± 0.22 and 0.534 ± 0.34 mg gallic acid equivalents per gram (mg GAE g^−1^) in treated fresh and dried samples, respectively) compared with untreated controls (1.264 ± 0.18 and 1.05 ± 0.67 mg GAE g^−1^). Similar reductions were observed for flavonoids and tannins. Antioxidant activity was also reduced, as indicated by values in the 2,2‐diphenyl‐1‐picrylhydrazyl (DPPH) assay and lower ferric reducing antioxidant power (FRAP) responses (*p* ≤ 0.001). These results suggest that BSF larvae feeding alters the chemical profile of *M. oleifera* residues; however, the compound‐specific alteration mechanism or larval accumulation was not assessed. Notably, reductions in secondary metabolites may represent a trade‐off between lowering anti‐nutritional factors and diminishing beneficial bioactive compounds. Future work employing targeted metabolomics and larval biomass analysis is needed to clarify the fate of individual phytochemicals. This study provides preliminary evidence for the potential use of BSF larvae in modifying plant residues for feed applications.

## 1 Introduction

*Moringa oleifera* Lam. (Moringaceae) is a fast‐growing tropical tree widely cultivated for its high protein content, vitamins, minerals, and bioactive compounds, making it a valuable resource for both human nutrition and livestock feed (Anwar and Bhanger 2003). Among its chemical constituents, *M. oleifera* leaves contain diverse secondary metabolites, including phenolics, flavonoids, tannins, saponins, and alkaloids. These compounds confer multiple bioactivities, such as antioxidant, antimicrobial, and anti‐inflammatory properties, while simultaneously serving as antinutritional or toxic factors that can reduce digestibility, nutrient absorption, and feed palatability in animals (Ramoza et al. 2024; Züst and Agrawal 2016). Consequently, strategies to modify or mitigate anti‐nutritional effects without compromising beneficial bioactivities are critical for sustainable feed utilization.

Black soldier fly (BSF; *Hermetia illucens* L., Diptera: Stratiomyidae) larvae have recently gained attention as bioconverters capable of transforming organic residues into larval biomass rich in protein and lipids for feed applications (Diener et al. 2011; Akwa and Gitau 2023). BSF larvae are non‐pest, non‐pathogenic, and tolerant of diverse substrates, suggesting potential utility for processing plant biomass containing secondary metabolites. Preliminary studies indicate that BSF larvae may alter the chemical composition of substrates through feeding and gut‐associated microbial activity, though direct evidence for specific metabolite degradation remains limited (Meijer et al. 2019; Kaprasob et al. 2017). Importantly, reductions in bulk secondary metabolites could represent a trade‐off: lowering anti‐nutritional compounds while potentially diminishing beneficial bioactive properties.

Despite its nutritional value, the suitability of *M. oleifera* leaves as a BSF substrate has not been fully investigated, particularly regarding the fate of secondary metabolites during larval feeding. Understanding how larval treatment influences the chemical and bioactive profile of *M. oleifera* residues is essential for evaluating feed safety and quality. This study therefore aimed to assess the effects of BSF larvae on bulk levels of secondary metabolites and antioxidant activity in fresh and air‐dried *M. oleifera* leaves, providing preliminary insights into the potential of BSF‐mediated modification of plant residues for feed applications.

## 2 Material and Methods

### 2.1 Collection and Authentication of *M. oleifera*

**FIGURE 1:**
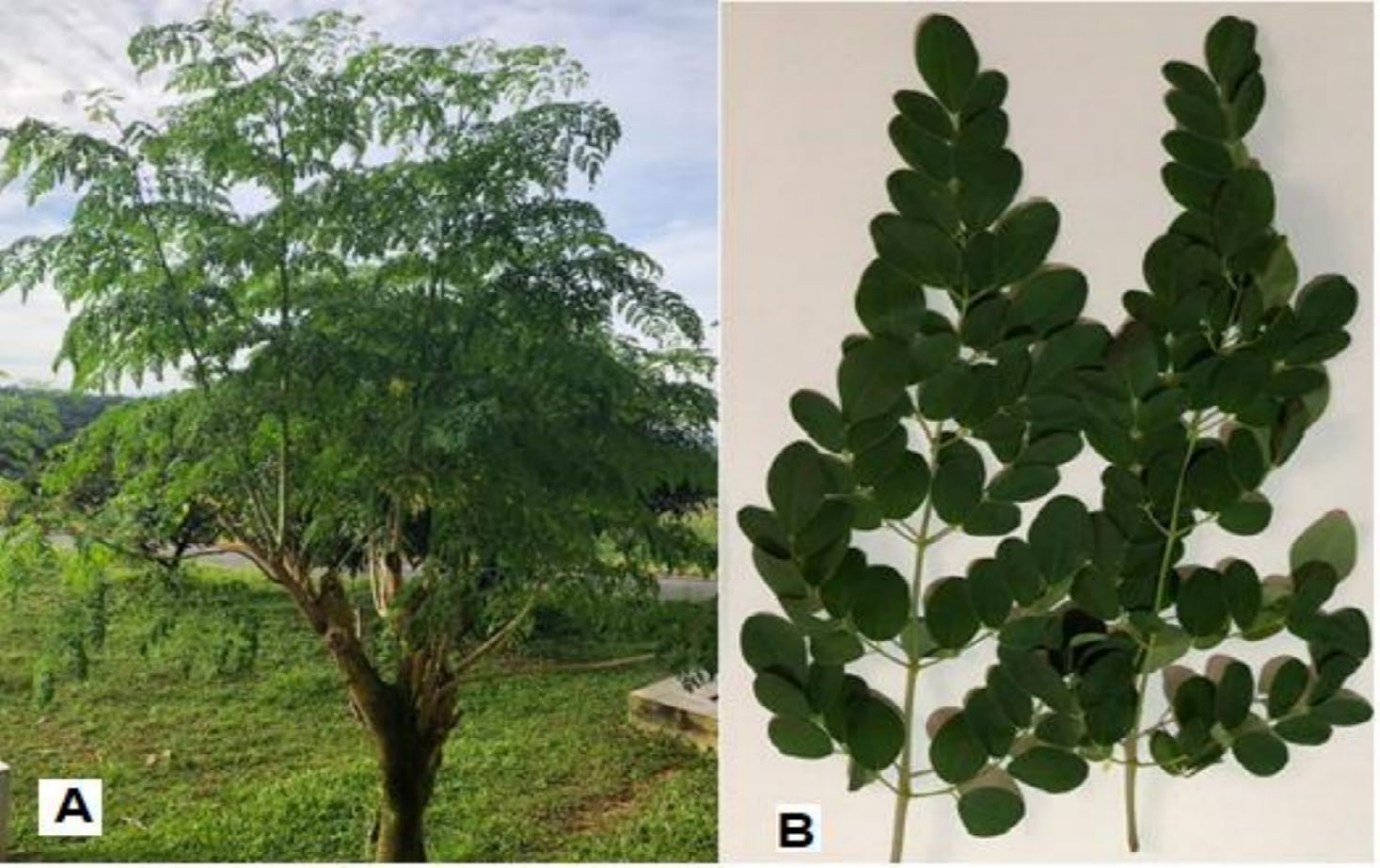
*Moringa oleifera* tree (A) and leaves (B). Photos taken by author during sample collection.

The *M. oleifera* leaves (Figure 1B) were harvested from the *M. oleifera* tree (Figure 1A) at the Institute of Development and Agricultural Research (IRAD) Dschang. This plant material was authenticated at the National Herbarium in Yaounde, Cameroon in comparison with voucher specimen deposited under the reference number 66896/HNC.

### 2.2 Sample Preparation

#### 2.2.1 Plant Preparation

Fresh leaves were chopped into small pieces to increase surface area for larval feeding. Air‐dried leaves were mixed with a minimal amount of water to facilitate larval interaction. Prepared samples (500 g per replicate) were distributed into plastic containers (height 14.0 cm; upper diameter 32.0 cm; lower diameter 23.5 cm). Each treatment was performed in three replicates.

#### 2.2.2 Black Soldier Fly Larvae Preparation

BSF ( *H. illucens* ) larvae originated from a colony maintained at the University of Dschang. Eggs were collected from wooden egg‐laying structures and reared on kitchen food waste until 7 days old under controlled conditions (27°C, 65% relative humidity, 16:8 h light: dark cycle). Larvae had an average individual weight of 22.41 ± 0.19 mg at 7 days of age.

### 2.3 Experimental Design

**FIGURE 2:**
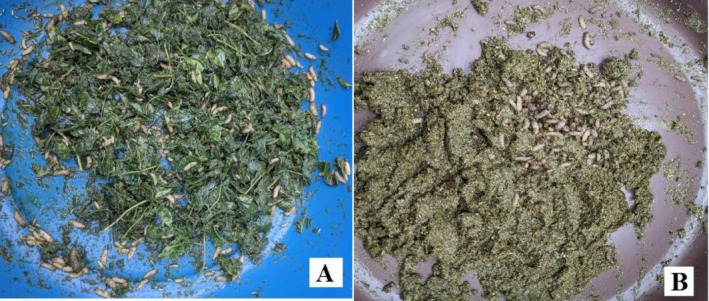
(A) Fresh *Moringa oleifera* leaves treated with black soldier fly larvae. (B) Air dried rehydrated *M. oleifera* leaves treated with black soldier fly larvae (photos taken by author during sample preparation).

Prepared *M. oleifera* samples, both fresh and air‐dried, rehydrated, were incubated with BSF larvae (Figure 2A,B) or without BSF larvae for 16 days under controlled conditions (27°C, 65% relative humidity, 12:12 h light: dark cycle). Containers were covered with mosquito nets to prevent contamination and allow airflow. Post‐treatment residues (mixture of residual substrate and frass) were collected, and larvae were sieved and separated manually.

### 2.4 Qualitative Analysis of *M. oleifera* Extracts

#### 2.4.1 Pretreatment of *M. oleifera* and Crude Extract Preparation

At the end of the 16th day period, plant material without the larvae and the residual plant material following larvae treatment were collected and transported to the laboratory, where approximately 300 g of it was macerated in 1500 mL of methanol for 48 h at room temperature, with occasional swirling. Filtrates were separated from residues using Whatman Number 1 filter papers and a vacuum pump. Liquids obtained were concentrated using a rotary evaporator at 64°C–65°C and 120 rpm and then allowed to air‐dry at room temperature. The obtained dry methanolic extract (crude extract) was weighed and stored at low temperatures (~5°C) for future use in the study.

#### 2.4.2 Phytochemical Screening of *M. oleifera* Extracts

A preliminary phytochemical assessment was performed to determine the baseline composition of *M. oleifera* extracts, following standard qualitative assays:

### 2.5 Flavonoids

The Shinoda test was used to detect flavonoids. Ten milligrams of extract were dissolved in 3 mL methanol and warmed, then 5 mg magnesium ribbon and 2 mL of 1% hydrochloric acid were added. The development of a pink or red coloration indicated the presence of flavonoids (Evans and Trease 2009).

### 2.6 Alkaloids

Alkaloids were identified using Mayer's test. Ten milligrams of extract were mixed with 3 mL of 50% aqueous hydrochloric acid, followed by three drops of Mayer's reagent (mercuric chloride and potassium iodide solution). A white or yellowish precipitate confirmed alkaloid presence (Evans and Trease 2009).

### 2.7 Saponins

The frothing test was employed for saponins. Ten milligrams of extract were dissolved in 5 mL distilled water, boiled for 5 min, cooled, and vigorously shaken for 30 s. Persistent foam exceeding 1 cm in height indicated saponins (Savithramma et al. 2011).

### 2.8 Triterpenes and Steroids

The Liebermann–Burchard test was applied. Ten milligrams of extract were dissolved in 3 mL chloroform, followed by 3 mL acetic anhydride, cooled on ice for 3 min, and a drop of concentrated sulfuric acid was added. Purplish‐red color indicated triterpenes, while blue, green, red, or orange color changes indicated steroids.

### 2.9 Anthraquinones

Ten milligrams of extract were dissolved in 4 mL ether–chloroform (1:1, v/v), then 4 mL of 10% sodium hydroxide were added. The appearance of a red color indicated quinones (Evans and Trease 2009).

### 2.10 Phenols

Phenolic compounds were detected by dissolving 10 mg of extract in 3 mL ethanol and adding three drops of 10% FeCl_3_. Blue‐violet or green coloration indicated the presence of phenols (Siddique et al. 2009).

### 2.11 Tannins

Ten milligrams of extract were boiled in 5 mL water, cooled, and mixed with 5 mL of 2% NaCl and 5 mL of 1% gelatin. Precipitate formation indicated tannins.

### 2.12 Anthocyanins

Ten milligrams of extract were boiled with 5 mL of 1% aqueous HCl. An orange color indicated anthocyanins.

### 2.13 Quantitative Determination of Secondary Metabolites

#### 2.13.1 Total Phenol Content

The total phenolic content of extracts was determined using the Folin–Ciocalteu method as described by Ramde‐Tiendrebeogo et al. (2012). The assay relies on the reduction of phosphotungstic–phosphomolybdic acid complexes by phenolic compounds to form blue oxides of tungsten and molybdenum, which absorb maximally at 765 nm (Luis et al. 2009). For each sample, 20 μL of extract (2 mg mL^−1^) was mixed with 100 μL of 10‐fold diluted Folin–Ciocalteu reagent and 80 μL of 20% sodium carbonate solution, incubated at 20°C for 30 min, and absorbance was measured at 765 nm. Blanks contained distilled water instead of extract. Gallic acid (0.015–2 mg mL^−1^) was used for calibration, and results were expressed as mg gallic acid equivalents per gram of extract (mg GAE g^−1^).

#### 2.13.2 Total Flavonoid Content

Flavonoids were quantified using the aluminum chloride colorimetric method (Chang et al. 2002). Each assay mixture contained 100 μL of extract (2 mg mL^−1^), 50 μL of 1.2% AlCl₃, and 50 μL of 120 mM potassium acetate, incubated at room temperature for 30 min. Absorbance was read at 415 nm. A calibration curve prepared with quercetin (0.015–2 mg mL^−1^) was used to calculate flavonoid content, expressed as mg quercetin equivalents per gram of extract (mg QE g^−1^).

#### 2.13.3 Total Tannin Content

Tannins were measured using the Folin–Ciocalteu method as described by Govindappa et al. (2011). Reaction mixtures included 100 μL of extract (2 mg mL^−1^), 500 μL of 10‐fold diluted Folin–Ciocalteu reagent, 1000 μL of 35% sodium carbonate, and 8.4 mL of distilled water. After 30 min incubation at room temperature, absorbance was read at 700 nm. A tannic acid calibration curve (100–500 μg mL^−1^) was used to express results as mg tannic acid equivalents per gram of extract (mg TAE g^−1^).

### 2.14 Evaluation of Antioxidant Activity

#### 2.14.1 DPPH Radical Scavenging Activity

The antiradical activity of extracts was evaluated using the DPPH assay (Mensor et al. 2001). In a 96‐well plate, 20 μL of extract (2 mg mL^−1^) was serially diluted across wells, followed by 180 μL of DPPH solution (0.08 mg mL^−1^) in the first three wells of each column. The fourth column received 180 μL of methanol as a blank. Plates were incubated in the dark for 30 min at room temperature, and absorbance was measured at 517 nm using a microplate reader. Vitamin C (l‐ascorbic acid) served as a positive control. Percent antioxidant activity was calculated using the formula:

$$\begin{array}{c} \%\text{Antioxidant activity}\\ =\frac{\left[\text{Absorbance of DPPH}-\left(\text{Absorbance of test}-\text{Absorbance of blank}\right)\right]\;}{\text{Absorbance of DPPH}\;}\\ \times 100 \end{array}$$

where Test = sample + methanolic DPPH solution and Blank = sample + methanol.

The different percentages of antioxidant activity were used for the determination of IC_50_ (the concentration of the sample capable of trapping 50% of DPPH) (Yassa et al. 2008). To do this, the regression lines were plotted using the values of the different percentages of antioxidant activity and the decimal logarithm of the sample. The equation of the regression line of the form *y* = *ax* + *b* was used. Assuming that *y* = 50, we obtain IC_50_ = 10^*x*^, where *x* = (50 − *b*)/*a*.

#### 2.14.2 Ferric Reducing Antioxidant Power (FRAP) Assay

The ferric reducing capacity of the extracts was assessed following Benzie and Strain (1996). The FRAP reagent was prepared by combining sodium acetate buffer (300 mM, pH 3.6), 10 mM TPTZ (2,4,6‐tris(2‐pyridyl)‐1,3,5‐triazine) solution, and FeCl₃ solution in a 10:1:1 ratio. A 5 μL aliquot of extract (2 mg mL^−1^) was mixed with 95 μL of FRAP reagent and incubated at 37°C in the dark for 30 min. Absorbance was measured at 593 nm using a FLUOstar Omega microplate reader. Vitamin C served as a positive control. Antioxidant activity was expressed as micromoles of FeSO_4_ equivalents per gram of extract, determined from a calibration curve prepared with FeSO_4_ (156.25–10,000 μM).

#### 2.14.3 Hydroxyl Radical Scavenging Assay

The capacity of extracts to inhibit hydroxyl radicals was determined according to Nagulendran et al. (2007). Reaction mixtures contained 60 μL FeCl_2_ (1 mM), 90 μL 1,10‐phenanthroline (1 mM), 2.4 mL phosphate buffer (0.2 M, pH 7.8), 150 μL H_2_O_2_ (0.17 M), and 1.5 mL of extract at concentrations of 12.5, 25, 50, 100, and 200 μg mL^−1^. Mixtures were incubated at room temperature for 5 min, and absorbance was read at 560 nm against a blank containing distilled water. Butylated hydroxytoluene (BHT) was used as a standard. Experiments were performed in triplicate, and higher absorbance values indicated greater hydroxyl radical scavenging activity.

### 2.15 Data Analysis

All measurements were performed in triplicate. Data were analyzed using R 4.2.3. One‐way ANOVA with substrate type as a fixed factor was performed, followed by Tukey's HSD for multiple comparisons. Statistical significance was set at *p* < 0.05. Data are reported as mean ± standard error (SE).

## 3 Results

### 3.1 Qualitative Phytochemical Composition

- Out of nine screened secondary metabolites, seven were detected in all samples (Table 1). Alkaloids, phenols, flavonoids, sterols, triterpenoids, tannins, and anthocyanins were present, while saponins and anthraquinones were absent in both treated and untreated *M. oleifera* samples. Larval treatment did not alter the qualitative presence of these compounds.

**TABLE 1:** Qualitative screening of secondary metabolites in *Moringa oleifera* extracts.

Extract	Alkaloids	Phenols	Flavonoids	Sterols	Triterpenoids	Tannins	Saponins	Anthocyanins	Anthraquinones
FMO	+	+	+	+	+	+	−	+	−
ADMO	+	+	+	+	+	+	−	+	−
FMOL	+	+	+	+	+	+	−	+	−
ADMOL	+	+	+	+	+	+	−	+	−

*Note:* Key: (+) detected, (−) not detected.

Abbreviations: ADMO = air‐dried *M. oleifera* leaves; ADMOL = air‐dried *M. oleifera* leaves + BSF larvae; FMO = fresh *M. oleifera* leaves; FMOL = fresh *M. oleifera* leaves + BSF larvae.

### 3.2 Quantitative Changes in Secondary Metabolites

Significant reductions (*p* ≤ 0.008) in secondary metabolite levels were observed following larval treatment (Table 2).

**TABLE 2:** Quantitative analysis of secondary metabolites in *Moringa oleifera* extracts.

Extracts	Total phenolics (mg GAE g^−1^)	Total flavonoids (mg QE g^−1^)	Total tannins (mg TAE g^−1^)
FMO	1.264 ± 0.18^b^	0.783 ± 0.22^c^	0.152 ± 0.006^b^
ADMO	1.05 ± 0.67^b^	0.356 ± 0.10^b^	0.104 ± 0.03^b^
FMOL	0.712 ± 0.22^a^	0.245 ± 0.01^b^	0.071 ± 0.02^a^
ADMOL	0.534 ± 0.34^a^	0.112 ± 0.23^a^	0.017 ± 0.01^a^
*p*	0.001	0.008	0.006

*Note:* Values are mean ± SE (*n* = 3). Different letters within a column indicate statistically significant differences at *p* < 0.05 (Tukey HSD).

Abbreviations: ADMO = air‐dried *M. oleifera* leaves; ADMOL = air‐dried *M. oleifera* leaves + BSF larvae; FMO = fresh *M. oleifera* leaves; FMOL = fresh *M. oleifera* leaves + BSF larvae; GAE = gallic acid equivalent; QE = quercetin equivalent; TAE = tannic acid equivalent.

Total phenols decreased from 1.264 ± 0.18 mg GAE g^−1^ in fresh untreated samples to 0.712 ± 0.22 mg GAE g^−1^ in larva‐treated fresh samples, and from 1.05 ± 0.67 mg GAE g^−1^ in dried untreated samples to 0.534 ± 0.34 mg GAE g^−1^ after treatment (*p* = 0.001).

Total flavonoids declined from 0.783 ± 0.22 mg QE g^−1^ (fresh untreated) to 0.245 ± 0.01 mg QE g^−1^ (treated fresh), and from 0.356 ± 0.10 mg QE g^−1^ (dried untreated) to 0.112 ± 0.23 mg QE g^−1^ (treated dried) (*p* = 0.008).

Total tannins were reduced from 0.152 ± 0.006 mg TAE g (fresh untreated) to 0.071 ± 0.02 mg TAE g (treated fresh), and from 0.104 ± 0.03 mg TAE g^−1^ (dried untreated) to 0.017 ± 0.01 mg TAE g^−1^ (treated dried) (*p* = 0.006). Overall, the order of metabolite concentration was:

$$\begin{array}{c} \text{Fresh untreated}\;\left(\mathrm{FMO}\right)>\text{dried untreated}\;\left(\text{ADMO}\right)\\ >\text{fresh treated}\;\left(\text{FMOL}\right)>\text{dried treated}\;\left(\text{ADMOL}\right) \end{array}$$

### 3.3 Antioxidant Activity

DPPH assay (IC_50_) values increased after larval treatment, indicating reduced antioxidant activity (Table 3). The IC50 values of FMO, ADMO, FMOL and ADMOL based on DPPH analysis were found to be significantly different (*p* = 0.001) and higher than that of the control. The antioxidant activity of larvae treated *M. oleifera* extracts was lower than that of the corresponding nontreated extracts. FMO had the highest antioxidant activity, followed by ADMO. A similar trend in results was observed from the FRAP analysis.

**TABLE 3:** Antioxidant activity of extracts: DPPH and FRAP results.

Extracts	DPPH test IC_50_ (μg mL^−1^)	FRAP Test (mmol FeSO_4_ g^−1^)
FMO	17.78 ± 0.45^b^	136.35 ± 0.45^c^
ADMO	73.11 ± 0.45^c^	96.79 ± 0.33^b^
FMOL	246.03 ± 0.43^d^	33.87 ± 0.56^a^
ADMOL	289.31 ± 0.3^d^	30.94 ± 0.58^a^
VIT C	2.43 ± 0.15^a^	158.36 ± 0.29^c^
*p*	0.001	< 0.001

*Note:* Values are mean ± SE (*n* = 3). Different letters indicate statistically significant differences at *p* < 0.05 (Tukey HSD).

Abbreviations: ADMO = air‐dried *Moringa oleifera* leaves; DPPH = 2,2‐diphenyl‐1‐picrylhydrazyl; FMO = fresh *M. oleifera* leaves; FRAP = ferric reducing antioxidant power.

### 3.4 Inhibition of Hydroxyl Radicals

Hydroxyl radical scavenging activity decreased in larva‐treated samples across all concentrations. Across the concentrations of the extracts decreased, so too was their absorbance. However, a significant decrease in absorbance was noted in the reaction mixture in the treatments containing larvae across their different concentrations (Table 4). This signified a decrease in the ability to reduce the hydroxyl radical in the treatments containing larvae. These results indicate a consistent reduction in hydroxyl radical inhibition capacity following larval treatment (*p* ≤ 0.006).

**TABLE 4:** Hydroxyl radical inhibition of *Moringa oleifera* extracts.

Extract	Concentration (μg mL^−1^)	200	100	50	25	12.5
FMO	Absorbance	0.514 ± 0.03^b^	0.257 ± 0.02^b^	0.137 ± 0.01^b^	0.126 ± 0.01	0.092 ± 0.01^a^
FMOL	Absorbance	0.442 ± 0.01^a^	0.221 ± 0.01^b^	0.122 ± 0.02^a^	0.110 ± 0.03	0.046 ± 0.02^b^
ADMOL	Absorbance	0.496 ± 0.02^a^	0.198 ± 0.01^b^	0.118 ± 0.02^a^	0.110 ± 0.01	0.045 ± 0.01^b^
ADMO	Absorbance	0.681 ± 0.01^b^	0.218 ± 0.02^b^	0.147 ± 0.01^b^	0.119 ± 0.03	0.102 ± 0.02^a^
BHT (control)	Absorbance	0.951 ± 0.02^c^	0.407 ± 0.03^a^	0.253 ± 0.02^c^	0.115 ± 0.01	0.111 ± 0.01^a^

*Note:* Values are mean ± SE (*n* = 3). Different letters within a concentration column indicate statistically significant differences at *p* < 0.05 (Tukey HSD).

Abbreviations: ADMO = air‐dried leaves; ADMOL = air‐dried leaves + BSFL; BHT = butylated hydroxytoluene; FMO = fresh leaves; FMOL = fresh leaves + BSFL.

## 4 Discussion

This study provides evidence that BSF ( *H. illucens* ) larvae alter the secondary metabolite profile of *M. oleifera* leaf substrates, as indicated by significant reductions in total phenolics, flavonoids, and tannins following larval treatment. One plausible explanation for the decline in secondary metabolites is the enzymatic activity associated with larval digestion and their gut microbiota. BSF larvae harbor diverse microbial communities capable of degrading complex organic compounds, including phenolics and tannins (Bruno et al. 2019). These compounds, which function as plant defense molecules, may be broken down into simpler metabolites or transformed into derivatives not detected by bulk spectrophotometric assays. However, it is important to recognize the limitations of the analytical approach in our current study. Our use of bulk extract‐based spectrophotometric assays quantifies broad classes of compounds rather than individual phytochemicals. Consequently, observed reductions likely reflect a combination of processes, including partial breakdown, structural modification (biotransformation), or compound sequestration (Mazlan et al. 2015; Meijer et al. 2019). Inference about specific metabolite alteration pathways therefore remains tentative, pending compound‐resolved analyses such as LC–MS/MS or metabolomic profiling.

Plant secondary metabolites are classically interpreted as defensive traits shaped by co‐evolution with herbivores. They deter feeding, reduce digestibility, or directly impair herbivore physiology through binding to digestive proteins or disrupting cellular processes (Makkar 2003). However, this functional characterization overlooks the broader ecological and biochemical roles of these compounds. Many phenolics and flavonoids also confer beneficial properties, such as antioxidant, anti‐inflammatory, and health‐promoting effects that are valued in human nutrition and agricultural applications (e.g., Moringa extracts exhibiting strong antioxidant capacity and diverse bioactivities). Plant secondary metabolites perform dual roles. While they can reduce digestibility and nutrient absorption acting as anti‐nutritional factors they also provide ecological and functional benefits, such as antioxidant, anti‐inflammatory, and antimicrobial activities (Ramoza et al. 2024; Kaprasob et al. 2017). This study provides preliminary evidence that BSF larvae treatment is associated with reductions in measured total phenolics, flavonoids, tannins, and antioxidant activity in *M. oleifera* residues. Across the bulk extract‐based assays, fresh leaves treated with larvae (FMOL) had total phenolics of 0.712 ± 0.22 mg GAE g^−1^ compared to 1.264 ± 0.18 mg GAE g^−1^ in untreated leaves (FMO), while air‐dried leaves treated with larvae (ADMOL) decreased to 0.534 ± 0.34 from 1.05 ± 0.67 mg GAE g^−1^ (ADMO). Similar trends were observed for flavonoids and tannins (Table 2). Antioxidant activity measured by DPPH IC_50_ and FRAP also declined following larval treatment, with FMOL showing IC_50_ = 246.03 ± 0.43 μg mL^−1^ compared to FMO = 17.78 ± 0.45 μg mL^−1^ (Table 3). These results indicate that BSF larvae feeding on *M. oleifera* residues can alter bulk chemical profiles, though whether this reflects enzymatic degradation, microbial biotransformation, metabolite binding in frass, or larval assimilation remains undetermined. The observed decrease in antioxidant activity, indicated by increased DPPH IC_50_ values and reduced FRAP, mirrors the reductions in total phenolics and flavonoids. Similar reductions in plant secondary metabolites during insect‐mediated bioconversion have been reported in organic waste processing systems (Diener et al. 2011; Makkar et al. 2014). Our current study thus highlights a critical trade‐off between reducing antinutritional factors and preserving beneficial phytochemicals. This duality underscores the need for optimizing BSF larvae treatment conditions, such as larval density and exposure duration, to achieve a balance between improved digestibility and retained bioactivity. Controlled bioconversion strategies have been proposed as a means to tailor substrate composition for specific feed applications (Barragan‐Fonseca et al. 2017).

### 4.1 Limitations to Findings and Future Work

While these observations are consistent with a reduction in bioactive secondary metabolites, several caveats must be considered. First, the posttreatment material analyzed consisted of a mixture of residual substrate and frass, which confounds chemical interpretation. Second, the analytical approach relied on bulk class‐based assays rather than compound‐specific quantification; thus, we cannot confirm which individual metabolites were degraded, transformed, or potentially accumulated within larvae. Third, larval biomass was not analyzed for metabolite content, leaving the possibility that reductions in residue levels reflect uptake rather than chemical transformation. Evidence suggests that some phytochemicals may be incorporated into insect tissues, potentially enhancing the nutritional or functional value of the larvae themselves (Liland et al. 2017). Finally, the study involved three replicates per treatment, which is typical for preliminary feeding assays, but observed variability (SE values in Tables 2, 3, 4) suggests that larger sample sizes may be necessary for robust statistical power.

Mechanistic hypotheses, such as enzymatic degradation or symbiotic gut microbial biotransformation, are plausible based on previous studies in insects (Meijer et al. 2019) but were not directly investigated here. Future work using targeted metabolomics, compound‐specific analyses, and characterization of larval gut microbiota is necessary to clarify which metabolites are degraded, transformed, or assimilated, and to assess the overall nutritional and bioactive profile of both residual substrate and larval biomass.

The observed decline in total secondary metabolite pools appears to reduce both anti‐nutritional and potentially beneficial bioactivities. This highlights a critical trade‐off between reducing anti‐nutritional factors and preserving beneficial phytochemicals. A balanced interpretation must also consider the potential trade‐offs associated with reduced secondary metabolites. Future work employing targeted metabolomics, mass balance studies, and assessments of downstream biological function will be essential to clarify the mechanistic and ecological consequences of larval bioconversion in applied and natural systems. Overall, this study provides a foundation for exploring BSF larvae as a tool for modifying plant residues for feed applications, emphasizing the importance of balancing reduction of antinutritional compounds with retention of beneficial phytochemicals to inform sustainable and safe feed production.

## 5 Conclusion

This study demonstrates that bioconversion of *M. oleifera* biomass using BSF larvae significantly reduces bulk secondary metabolites, including phenolics, flavonoids, and tannins, alongside a corresponding decline in antioxidant activity. These findings indicate that larval processing alters the chemical composition of plant material in ways that may enhance its suitability as animal feed by lowering antinutritional factors known to impair digestibility and palatability. However, the concurrent reduction in antioxidant capacity highlights an important trade‐off, as beneficial bioactive compounds are also diminished. This dual effect underscores the need to carefully optimize larval treatment conditions to balance improved nutritional quality with retention of functional phytochemicals. Importantly, the study provides only a bulk‐level assessment and does not clarify the specific pathways or fate of individual metabolites. Whether these compounds are degraded, transformed into other bioactive forms, or accumulated within larval biomass remains unresolved. Addressing these gaps through targeted metabolomics and analysis of larval tissues will be essential for a more comprehensive understanding of the bioconversion process. Overall, the results provide preliminary evidence supporting the use of BSF larvae as a sustainable tool for modifying plant residues for feed applications. Future research should focus on process optimization, compound‐specific transformations, and the nutritional implications for both the treated biomass and the resulting insect‐derived products.

## Funding

This study was carried out with funds from the DAAD (German Academic Exchange Service) program for Sub‐Saharan Africa granted to the first and corresponding author.

## Conflicts of Interest

The authors declare no conflicts of interest.

## Data Availability

The datasets generated and analyzed during the current study have been deposited in the Zenodo repository with the reserved DOI 10.5281/zenodo.19553930. The repository contains all raw experimental measurements, including total phenolics, flavonoids, tannins, DPPH IC_50_, and FRAP values across all treatments and biological replicates, as well as associated metadata describing the experimental design, treatment structure, and variable definitions. In addition, the R script (analysis_script.R) used to perform all statistical analyses is provided to ensure full transparency and reproducibility of the results.
